# 977. Early versus Delayed Physical Therapy in Burn Patients: A Propensity-matched Analysis

**DOI:** 10.1093/jbcr/irag033.574

**Published:** 2026-04-06

**Authors:** Joshua Wang, Philong Nguyen, Sudhanvan Iyer, Kamryn K Baker, Joshua Borja, Noelle Gonzalez, Yousef Tanas

**Affiliations:** University of Texas Medical Branch, Galveston, TX; University of Texas Medical Branch, Galveston, TX; University of Texas Medical Branch, Galveston, TX; McGovern Medical School at University of Texas Health Science Center at Houston, Houston, TX; University of Texas Medical Branch, Galveston, TX; University of Texas Medical Branch - John Sealy School of Medicine, Houston, TX; Houston Methodist Hospital, Houston, TX

## Abstract

**Introduction:**

The timing of physical therapy (PT) initiation in burn patients is controversial. While early PT is often advocated to reduce contractures and improve recovery, the risks and benefits compared to delayed initiation remain unclear. Understanding the impact of PT timing on both short- and long-term outcomes is critical for optimizing burn rehabilitation strategies.

**Methods:**

The TriNetX Research Network was queried for patients ≥18 years old with burn injuries between 2010 and 2023. Patients who received early PT were matched 1:1 to those who received delayed PT using propensity score matching. Matching variables included demographics, body mass index, comorbidities, substance use, and total burn surface area. The primary outcome was hypertrophic scarring. Secondary outcomes included infection, wound disruption, sepsis, deep vein thrombosis (DVT), and pulmonary embolism (PE). Outcomes were assessed at 90 days, 6 months, and 1 year. Hazard ratios (HRs) with 95% confidence intervals (CIs) were calculated using Cox proportional hazards models.

**Results:**

After propensity score matching, 7238 patients were included (n = 3619 per cohort). At 90 days, there were no significant differences in infection, wound disruption, or DVT. Delayed PT was associated with lower risk of hypertrophic scarring (HR 0.33, 95% CI 0.25–0.44, *p*<.001), sepsis (HR 0.67, 95% CI 0.52–0.87, *p*=.002), and PE (HR 0.64, 95% CI 0.44–0.91, *p*=.013). At 6 months, delayed PT continued to show reduced scarring (HR 0.31, 95% CI 0.25–0.39, *p*<.001), sepsis (HR 0.79, 95% CI 0.63–0.98, *p*=.034), and PE (HR 0.61, 95% CI 0.44–0.85, *p*=.003). At 1 year, infection, wound disruption, sepsis, and DVT were not significantly different, but scarring (HR 0.33, 95% CI 0.27–0.40, *p*<.001) and PE (HR 0.73, 95% CI 0.54–0.97, *p*=.032) remained significantly lower with delayed PT.

**Conclusions:**

Early initiation of PT in burn patients was not associated with improved outcomes and was linked to higher risks of scarring, sepsis, and pulmonary embolism. Delayed PT demonstrated more favorable results across multiple timepoints, suggesting that timing of PT should be individualized rather than universally accelerated in burn rehabilitation protocols. However, future prospective studies are required to further elucidate this relationship.

**Applicability of Research to Practice:**

These findings challenge the common assumption that early initiation of physical therapy is universally beneficial for burn patients. The data suggest that delaying PT may reduce risks of hypertrophic scarring, sepsis, and pulmonary embolism, without increasing wound complications or thromboembolic events. In practice, this supports a more individualized approach to rehabilitation, where the timing of PT initiation is guided by patient stability, wound healing, and overall risk profile rather than a standardized early-start protocol.

**Funding for the study:**

N/A.

